# A mouse model featuring tissue-specific deletion of p53 and Brca1 gives rise to mammary tumors with genomic and transcriptomic similarities to human basal-like breast cancer

**DOI:** 10.1007/s10549-018-5061-y

**Published:** 2018-11-27

**Authors:** Daniel P. Hollern, Cristina M. Contreras, Stephanie Dance-Barnes, Grace O. Silva, Adam D. Pfefferle, Jessie Xiong, David B. Darr, Jerry Usary, Kevin R. Mott, Charles M. Perou

**Affiliations:** 1 Lineberger Comprehensive Cancer Center, University of North Carolina at Chapel Hill, 450 West Drive, CB#7264, Chapel Hill, NC 27599 USA; 2 Department of Genetics, University of North Carolina, Chapel Hill, NC 27599 USA; 3 Department of Biological Sciences, Winston Salem State University, Winston-Salem, NC 27110 USA; 4 Arrow Genomics LLC, Chapel Hill, NC 27517 USA

**Keywords:** Breast cancer, Mouse models, Chemotherapy, Genomics, Copy number, Immune cells

## Abstract

**Purpose and methods:**

In human basal-like breast cancer, mutations and deletions in TP53 and BRCA1 are frequent oncogenic events. Thus, we interbred mice expressing the CRE-recombinase with mice harboring loxP sites at TP53 and BRCA1 (K14-Cre; p53^f/f^ Brca1^f/f^) to test the hypothesis that tissue-specific deletion of TP53 and BRCA1 would give rise to tumors reflective of human basal-like breast cancer.

**Results:**

In support of our hypothesis, these transgenic mice developed tumors that express basal-like cytokeratins and demonstrated intrinsic gene expression features similar to human basal-like tumors. Array comparative genomic hybridization revealed a striking conservation of copy number alterations between the K14-Cre; p53^f/f^ Brca1^f/f^ mouse model and human basal-like breast cancer. Conserved events included MYC amplification, KRAS amplification, and RB1 loss. Microarray analysis demonstrated that these DNA copy number events also led to corresponding changes in signatures of pathway activation including high proliferation due to RB1 loss. K14-Cre; p53^f/f^ Brca1^f/f^ also matched human basal-like breast cancer for a propensity to have immune cell infiltrates. Given the long latency of K14-Cre; p53^f/f^ Brca1^f/f^ tumors (~ 250 days), we created tumor syngeneic transplant lines, as well as in vitro cell lines, which were tested for sensitivity to carboplatin and paclitaxel. These therapies invoked acute regression, extended overall survival, and resulted in gene expression signatures of an anti-tumor immune response.

**Conclusion:**

These findings demonstrate that this model is a valuable preclinical resource for the study of human basal-like breast cancer.

**Electronic supplementary material:**

The online version of this article (10.1007/s10549-018-5061-y) contains supplementary material, which is available to authorized users.

## Introduction

Breast cancer is the second leading cause of cancer-related deaths in American women [1]. Clinically, breast cancer is a heterogeneous disease with multiple histological subtypes, differences in patient outcomes, and differential expression of critical tumor biomarkers [2]. Genomic characterization of human breast tumors has resulted in the identification of five distinct tumor subtypes: Luminal A and B, HER2-enriched, Claudin-low, and Basal-like. These five subtypes have their own unique biology, which correlates with distinct patient outcomes [3]. About half of the cases of hereditary breast cancer have germ-line mutations in the BRCA1 gene, and BRCA1 mutant tumors commonly possess mutations in the TP53 tumor suppressor gene [4]. These BRCA1 mutant tumors often lack expression of the estrogen, progesterone, and HER2 receptors (termed “triple-negative”) and possess a basal-like intrinsic gene expression phenotype/subtype [5].

Previous studies comparing genetically engineered mouse models (GEMMs) with human breast cancer identified GEMMs that distinctly represent individual human breast tumor subtypes at the gene expression level [6–8]. Developing GEMMs that mimic specific human breast cancer subtypes is vital for the translation of preclinical results into effective human clinical trials. Given that BRCA1 and TP53 loss is a common occurrence in basal-like breast cancer, we hypothesized that the deletion of these genes in the mammary epithelium would give rise to tumors with basal-like features. Thus, we interbred mice with loxP sites flanking critical exons of the BRCA1 and Tp53 genes with mice hemizygous for Cre under the control of the Keratin-14 promoter. The resulting mouse model was termed KPB1 (short for K14-cre; p53^f/f^ Brca1^f/f^). Here, we provide a multi-platform analysis to credential this mouse model for the study of human breast cancer. Importantly, we detail primary tumors, cell lines, and tumor transplant lines using gene expression profiling, DNA copy number analysis, and sensitivity to chemotherapy.

## Materials and methods

### Transgenic mice

K14-cre mice (FVB-Tg(KRT14-cre)8Brn/Nci) were obtained from the Mouse Models of Human Cancers Consortium (Strain: 01XF1). The Brca1 and p53 double lox/lox mutation was developed by Karl Simin and Terry Van Dyke using strains FVB;129-Brca1tm1Brn/Nci and FVB.129P2-Trp53tm1Brn/Nci and were bred onto the FVB background. Athymic nude mice (No: 002019) and FVB mice (No: 001800) were obtained from the Jackson Laboratory. Work was performed in accordance with approved University of North Carolina (UNC) Institutional Animal Care and Use protocols.

### Serial tumor passaging

Tumors were digested in collagenase/hyaluronidase for 1 h at 37 °C. Cell aggregates were washed with Hank’s Balanced Salt Solution containing 2% FBS and suspended in HF media with 50% Matrigel™. Mice were briefly anesthetized with 2% isoflurane and tumor cells were injected into the inguinal mammary fat pad.

### Immunohistological and immunofluorescent analyses of mouse tumor tissue

Tumors were fixed in 10% formalin (Sigma-Aldrich) overnight and washed in 70% ethanol. Paraffin-embedded sections were processed by the UNC Animal Histopathology Core. Routine hematoxylin and eosin (H&E) staining was performed. For immunofluorescence, the antibodies and dilutions were α-CK-5 (1:8,000, PRB-160P, Covance), and α-CK-8/18 (1:450, GP11, Progen Biotecknik). Heat-mediated epitope retrieval was performed in boiling citrate buffer (pH 6.0) for 15 min, samples were cooled to room temperature for 30 min. Secondary antibodies for immunofluorescence were conjugated with Alexa Fluor-488 or Alexa Fluor-594 (1:200, Molecular Probes, Invitrogen). Immunofluorescence samples were mounted with VectaShield Hardset using DAPI mounting media (Vector).

### Gene expression microarrays

RNA processing and labeling was done as described [7]. Equal quantities of labeled mouse reference cDNA and tumor cDNA were co-hybridized overnight to Agilent 4×180K whole mouse genome microarrays. We incorporated 46 new KPB1 samples for comparative analysis to a previously published dataset of 27 genetically engineered mouse models [7]. The 63 chemotherapy treated samples were analyzed separately. Samples were normalized as described [9]. New gene expression data can be found in GEO under GSE122076. For comparisons to human breast cancer, we used the TCGA breast dataset. Count data were obtained using STAR alignment and SALMON quantification. Upper quartile normalization was applied. Genes with an average expression of less than three were removed, and the data were then log2 transformed. For TCGA and murine datasets, genes present in less than 70% of the samples were removed. Genes were median centered and samples were column standardized. The murine dataset was aligned to HGNC gene symbols and combined with the TCGA dataset using COMBAT [10]. Gene expression signatures were calculated as published [11, 12].

### Copy number determination

Genomic DNA was labeled using the Agilent Genomic DNA Labeling Kit PLUS (Cat# 5188–5309), co-hybridized with normal reference DNA to 2×244K Mouse aCGH Agilent arrays (Cat # G4415A), and scanned on an Agilent DNA Microarray scanner. Intensity files were uploaded to the University of North Carolina Microarray Database (http://www.genome.unc.edu) for LOWESS normalization. SWITCHdna and SWITCHplus [13] software was used to identify significantly altered segments, annotate segments, and map cross-species alterations. Published “UNC159” tumor data are available on the Gene Expression Omnibus (GEO) under accession GSE52173. Level 3 data for TCGA samples were downloaded from the Genomic Data Commons data portal. Human data were processed as described [13]. The published murine datasets [13, 14] are under GEO accessions GSE27101 and GSE52173. The 21 new murine samples (18 KPB, 3 MMTV-PyMT) are deposited under GEO accession GSE122076.

DawnRank was used to predict drivers; it is a novel computational method to integrate predetermined protein–protein interaction network and gene expression data within individuals to rank genes [12, 13, 15]. Genes that have a high differential expression as well as a high degree of perturbation of downstream genes in the network receive a high score. Potential driver genes for a set of samples were then determined by aggregating individual DawnRank gene scores using a Condorcet voting scheme integrating copy number data. Briefly, genes that frequently have high DawnRank scores and are copy number altered in the samples rank high in the final driver list.

### Treatments

When tumors reached 5 mm in one dimension, the mice were randomized into treatment or control groups. Tumor-bearing mice were treated with carboplatin (50 mpk) and paclitaxel (10 mpk) once per week. Mice were observed for overall condition and weighed bi-weekly. Mice that developed adverse side effects were removed from study. Therapeutics were obtained from commercial sources: Carboplatin (Hospira) and Paclitaxel (Ivax Pharmaceuticals).

## Results

### Latency and histopathological features of KPB1 tumors

**Fig. 1:**
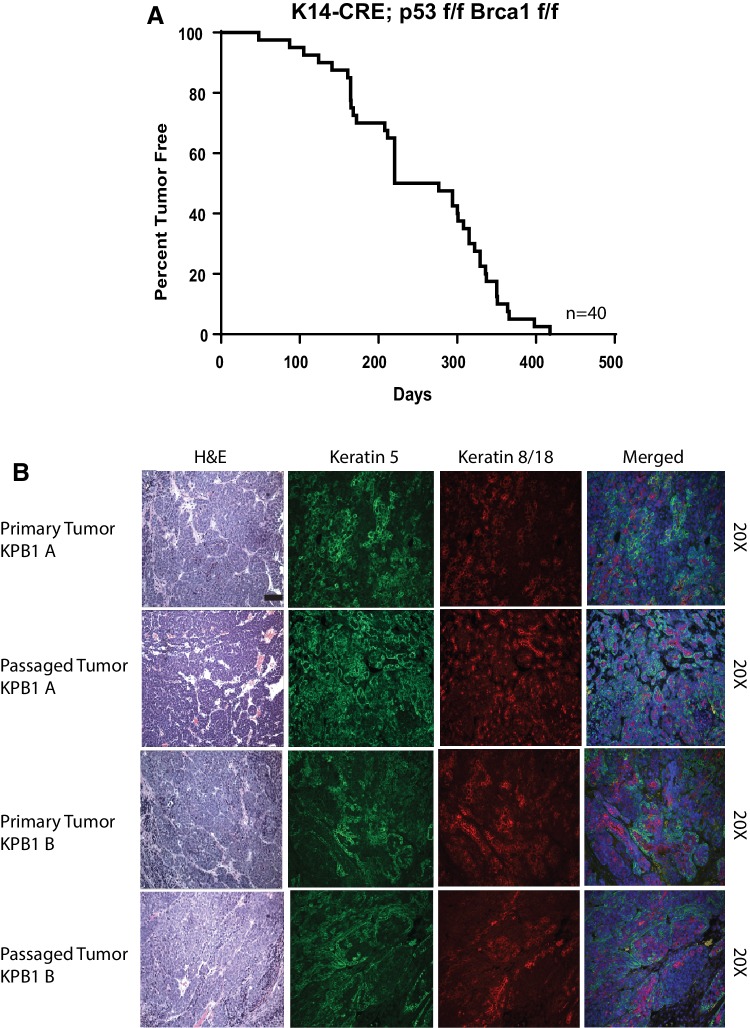
Phenotypic assessment of K14-Cre; p53^f/f^ Brca1^f/f^ tumors features long tumor latency and expression of basal cytokeratins. **a** Percent tumor-free survival (tumor latency) for K14-Cre; p53^f/f^ Brca^f/f^ tumors. **b** Hematoxylin and eosin staining, keratin-5, and keratin-8/18 staining is shown for parent KPB1 primary tumors and the tumor transplant lines derived from the primary tumors. Photos are shown at ×20 magnification

K14-cre; P53^f/f^ Brca1^f/f^ male mice were bred with P53^f/f^;Brca1^f/f^ female mice. Pups were born at the expected litter sizes with the correct distribution of genes, demonstrating that this cross is not embryonic lethal. The KPB1 offspring mice had alopecia, developed lesions on their skin, had poor grooming habits, and frequently acquired dental malocclusions. Other tumors that arose included skin, eye, and possibly lymphomas; these were not further investigated. Of the females surviving without lesions that required euthanasia, 85% developed mammary tumors. Median survival of mice that did develop mammary tumors was 248.5 days (Fig. 1a).

### Histopathological features of KPB1 tumors

Mammary tumors were highly cellular and densely packed with stroma nearly absent (Fig. 1b). Tumors were poorly differentiated in appearance, forming nests of cells with abundant mitotic figures. Because of the long tumor latency, we serially passaged tumors into female nude mice and also into syngeneic wild-type FVB females. Tumors grew in both types of mice and all subsequent passages were performed on FVB females to preserve the intact mouse immune responses. This established the KPB1A and KPB1B transplant lines. Histologically, the passaged tumors were very similar to the original parent tumors in both the cellularity and comparable number of tumor nests that were observed. Both primary and passaged tumors expressed similar levels of both Keratin 5 and Keratin 8/18 (Fig. 1b), as is observed in basal-like breast cancer [16, 17].

### KPB1 transgenic mice develop basal-like mammary tumors

**Fig. 2:**
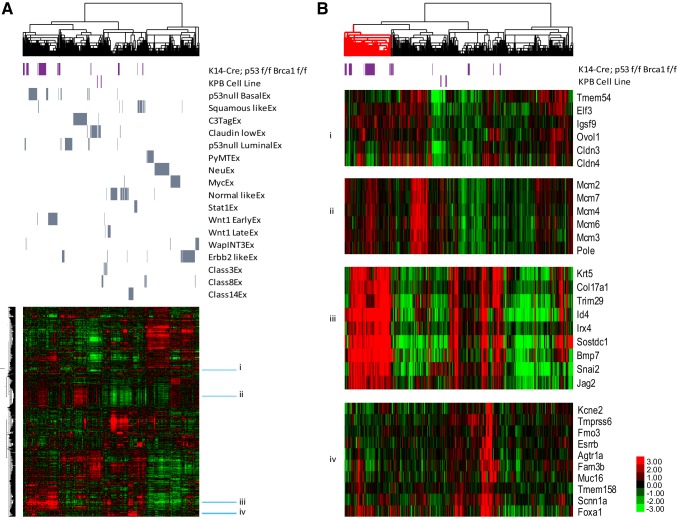
Intrinsic analysis of K14-Cre; p53^f/f^ Brca1^f/f^ tumors reveals basal-like gene expression profiles. **a** Hierarchical clustering using centroid linkage to analyze the relationship of KPB1 tumors to other GEMMs using an intrinsic gene set [7]. Across the top, the dendrogram shows the similarity between samples based on gene expression values. The purple bars highlight the position of KPB1 tumors and cell lines in the dendrogram and the heatmap below. Similarly, gray bars itemize the position of tumors from the corresponding gene expression class (as published, [7]). Beside the heatmap, sky blue bars depict clusters highlighted in **b. b** Selected clusters from **a** show expression of key intrinsic gene clusters as follows: (i) genes that identify Claudin-low tumors, (ii) genes associated with cell proliferation, (iii) genes that identify basal-like tumors, and (iv) genes that identify luminal-like tumors. All heatmaps are color coded according to the scale shown in bottom right-hand corner. Prior to clustering, data were pre-processed as described in the methods and then filtered to intrinsic genes

RNA-expression arrays were run on 35 primary mammary tumors. In addition, we arrayed replicates of the tumor transplant lines and cell lines created from KPB1 tumors. These samples were combined with a published dataset of 27 GEMMs and then clustered using a mouse intrinsic gene list that is used to define murine mammary expression subtypes (Fig. 2a) [7]. Of the primary KPB1 tumors, the majority clustered with other basal-like tumors (i.e., p53 Null basal-like). Importantly, the KPB1A and KPB1B tumor transplant lines clustered beside their parent tumors in this main basal-like cluster. Another subset of KPB1 tumors clustered with squamous tumors; this is noteworthy as squamous tumors also possess basal-like features [18]. KPB1 cell lines clustered with Claudin-low tumors. Using SigClust [19], we found the main cluster of basal-like tumors to be statistically distinct from other clusters (*p* < 0.05, Fig. 2b, red cluster). Molecularly, this cluster of KPB1 tumors had high expression of basal cell markers, low expression of luminal markers, and high expression of proliferation markers (Fig. 2b).

To test for similarities to human breast cancer, we combined our murine dataset with the TCGA breast cancer dataset. Using clustering analysis and an intrinsic subtypes gene list [7], the majority of the KPB1 tumors clustered with human basal-like tumors (Fig. S1A). Importantly, the KPB1 tumors matched human basal-like tumors for high expression of basal-associated genes, low expression of luminal-like genes and claudin genes, and high expression of proliferation genes (Fig. S1B). Together, these results depict some diversity amongst tumors in this KPB1 model; however, the main tumor outcome was transcriptionally basal-like. As a result, we focused on analyzing these basal-like KPB1 tumors (Fig. 2b, red cluster) in more detail with array-CGH (aCGH).

### Copy number alterations are conserved between basal-like tumors

**Fig. 3:**
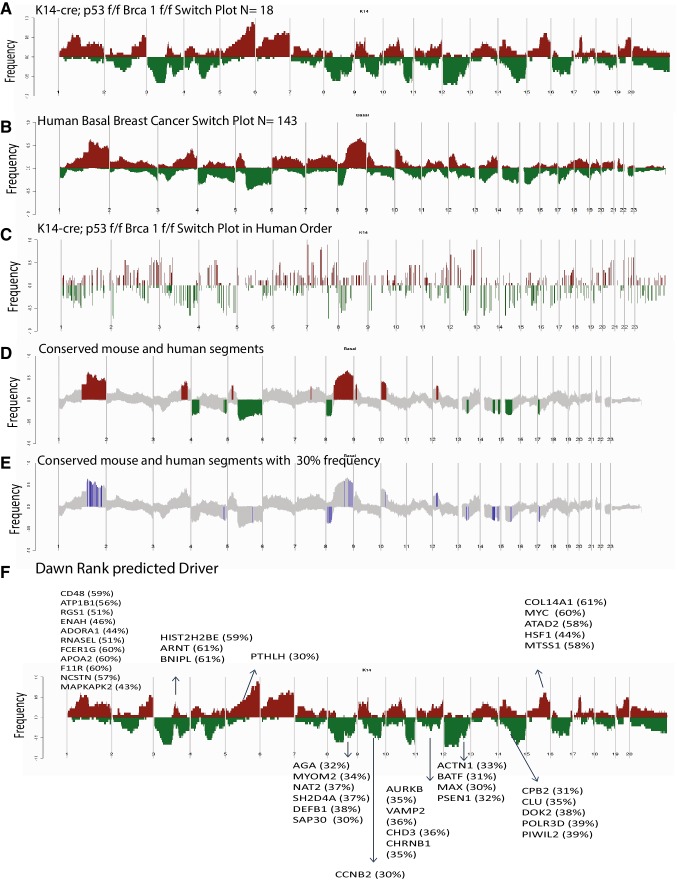
Analysis of K14-Cre; p53^f/f^ Brca1^f/f^ tumors reveals conserved copy number alterations with basal-like human breast cancer. **a** Gains (red) and losses (green) are shown for the KPB1 murine tumors, **b** human basal breast tumors, **c** the KPB1 murine tumors with gains/losses shown in human chromosome order. **d** All conserved gains/losses between murine KPB1 tumors and human basal-like tumors are shown. **e** Conserved gains and losses with a 30% or greater frequency in both KPB1 and human basal-like tumors are shown. **f** The mapping of driver mutations predicted by the DawnRank algorithm is shown on the KPB1 murine switch plot from **a**. For all panels, the frequency of alterations in each group is indicated on the *y*-axis, where the frequency of losses is shown from 0.0 to − 1.0 (for example, a value of − 0.5 indicates loss in 50% of samples) and frequency of gains is shown from 0.0 to 1.0

The transcriptional similarities of murine KPB1 tumors to human basal-like tumors suggested that copy number alterations (CNAs) may also be shared, thus we used aCGH to test for common areas of copy number amplification and deletion across species. The DNA copy number landscape of the KPB1 tumors (Fig. 3a) and human basal-like tumors (Fig. 3b) showed many reoccurring CNAs. To facilitate the discovery of conserved changes, we remapped KPB1 frequency landscape plots in human chromosome order [13] (Fig. 3c). Overlapping the remapped KPB1 CNAs frequency landscape onto the TCGA basal-like plot revealed a significant degree of shared CNAs in KPB1 and human basal-like tumors (Fig. 3d). As shown in Fig. 3d, human basal-like events also found in the KPB1 model included gains at Chr 1q, Chr 3q, Chr 5p, Chr 8q, Chr 10p, and Chr 12p. Common losses were observed at Chr 4, Chr 5q, Chr 8p, Chr 13p, Chr 14q, Chr 15p, and Chr 17p. Next, we examined copy number gains or losses occurring with 30% frequency in both human basal-like and KPB1 tumors (highlighted blue in Fig. 3e). In total, almost 400 genes were common as gained or lost in both human and mouse tumors; thus many copy number changes were conserved across species.

To determine which of the conserved copy number gains and losses were genomic drivers in the formation of these tumors, we ran expression data and aCGH data through DawnRank analysis [15]. DawnRank is a program that integrates DNA changes, with RNA expression and projected into protein–protein interaction networks, to find those genetic alterations that are the most changed using RNA expression. We then took the top 20% of the DawnRank gene output and overlapped it with our human/mouse conserved list of 400 copy number altered genes. The top 20 common genes gained and lost were selected as genetic drivers and included known genes such as MYC, HSF1, PSEN1, and NCSTN (Nicastrin). These results are highlighted on the mouse landscape to show events that correspond to predicted drivers (Fig. 3f).

**Fig. 4:**
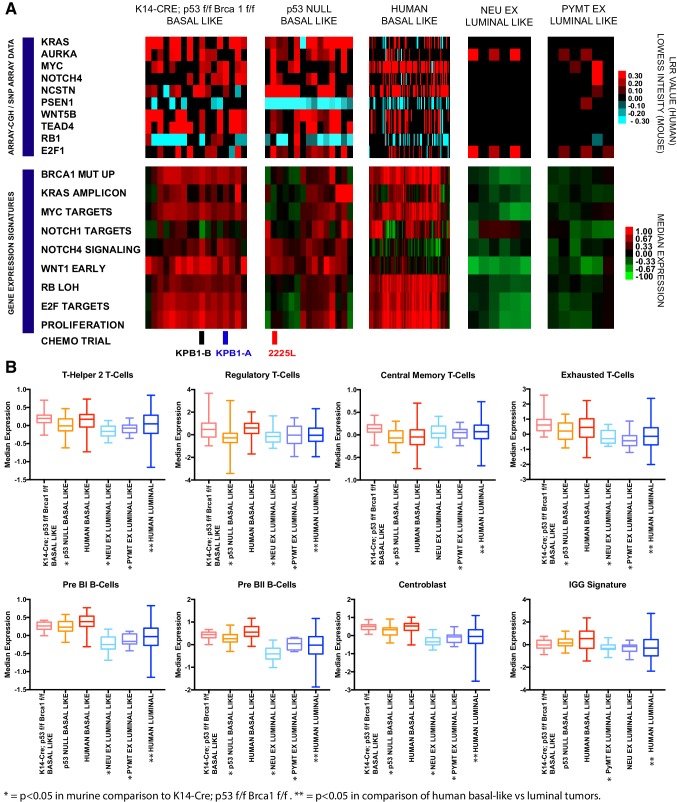
Examination of conserved features amongst basal-like tumors. **a** Array-CGH LOWESS intensity data are shown for murine models (KPB1 basal-like *n* = 18, p53 Null basal-like *n* = 15, Neu^Ex^*n* = 7 and PyMT^Ex^*n* = 6) with matching microarray data. Similarly, SNP-array LRR values are shown for human basal-like samples with matching RNA-seq data (*n* = 88). All data processing for array-CGH and SNP arrays are as published [13] and normalized intensity values (LOWESS or LRR) are shown according to the blue–black–red color bar. Below the copy number data, heatmaps for gene expression signatures are shown in matched samples. All signatures are previously publicly available as follows: Brca1 mut up [20], Kras amplicon [11], Myc targets [21], Notch1 targets [22], Notch4 signaling (molecular signatures database [23], reactome signaling by notch 4), Wnt1 early [7], RB LOH [24], E2F Targets [25], and Proliferation [11]. **b** Box and whiskers plots of immune cell signatures across murine (KPB1-basal-like *n* = 31, p53 basal-like *n* = 33, Neu^Ex^*n* = 36, and PyMT^EX^*n* = 17) and human tumors (basal-like *n* = 136, and luminal-like *n* = 591). All immune cell signatures are previously published as follows: Th2 T-cells [26], Regulatory T-cells [26], Central memory T-cells [26], Exhausted T-cells [27], Pre BII and Pre BII B-cells [28], Centroblasts [29], and IGG signature [11]. *t* tests were unpaired and two-tailed *p* values are reported as follows: *p < 0.05 for KPB1 model compared to corresponding mouse models; **p < 0.05 for human basal-like versus luminal-like tumors

Amongst frequently altered genes, we noted cooperative events in several key oncogenic pathways. This included common human and murine alterations in the Myc pathway (KRAS, AURKA, MYC), the Notch pathway (NOTCH4, NCSTN, PSEN1), the Wnt pathway (WNT5B, TEAD4), and the RB pathway (RB1 (loss), E2F1 (gain)). We observed that many of these events are conserved in the basal-like tumors but are not common in luminal mouse models (Fig. 4a). Matched RNA-expression profiling and gene expression signatures provided evidence that these events impart corresponding pathway activation. For example, the KPB1 model showed high expression of the BRCA1 mutant signature; this signature was also elevated in human basal-like tumors. The gains in KRAS, AURKA, and MYC corresponded to high expression of the KRAS amplicon signature and high expression of c-Myc target genes in KPB1 tumors and many of the human basal-like tumors. The events in the Notch pathway corresponded to the elevation of Notch pathway signatures. Finally, RB1 deletions were frequent in the murine and human basal-like tumors and corresponded to high proliferation signatures as expected. Together, these data show that many of the copy number alterations found in basal-like breast cancers (both murine and human) impact pathway activation and the transcriptional profiles present in tumors.

### KPB1 tumors show immune infiltration

A key component of tumor biology is the immune microenvironment. Therefore, we tested tumors for key immune cell types using gene expression signatures (Fig. 4b). KPB1 tumors presented distinct patterns for T-cell signatures, showing higher expression of T-helper 2 (Th2) ,T-regulatory, central memory, and exhausted T-cell signatures (*p* < 0.05). Human basal-like tumors shared an elevation of these signatures as well. Consistent with the ‘immune suppressive’ nature of some of these signatures, KPB1 and human basal-like tumors also shared Pd1 and Ctla4 signaling patterns (Fig. S2). PD-L1 was elevated in human basal-like tumors and had moderate expression KPB1 tumors. Significant changes in the signatures of B-cells were also detected; Pre BI, Pre BII, and centroblast signatures were all higher in KPB1, p53^−/−^ basal-like, and human basal-like tumors (*p* < 0.05). In addition, a signature for immunoglobulin G (IGG) was also elevated in human basal-like tumors. In the KPB1 model, this signature was significantly higher than in MMTV-PyMT tumors. Despite this, B-cell activation signatures were not highly expressed in KPB1 tumors (data not shown). Thus, B-cells are likely present, but may not be activated. Collectively, these data provide evidence that KPB1 tumors share many key immunological features with human basal-like tumors, featuring T-cell and B-cell infiltration and an overall immune-suppressed microenvironment.

### KPB1 tumors are sensitive to DNA-damaging agents

**Fig. 5:**
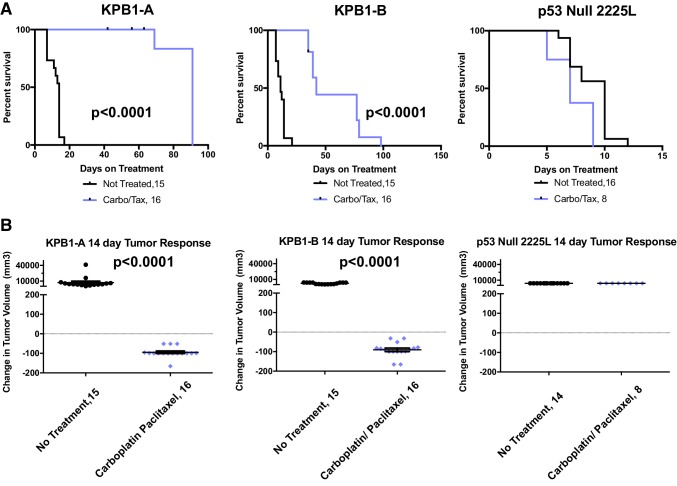
Testing sensitivity to carboplatin–paclitaxel combination chemotherapy in tumor transplant lines. **a** Long-term survival is shown by Kaplan–Meier plots for syngeneic recipient mice receiving KPB1A tumors, KPB1B tumors, or 2225L p53 Null tumors. Mice bearing 5 mm tumors were randomly assigned to the no-treatment control group (black lines) or the therapeutic arm where mice receive a combination therapy of carboplatin (50 mpk) and paclitaxel (10 mpk) once per week (purple lines). Mice were euthanized when tumors reached 20 mm in the largest diameter. **b** 14-day acute response is shown for KPB1A, KPB1B, and 2225L recipient mice. *p* values are two-tailed and reflect the results of unpaired *t* tests

Human BRCA1 mutant tumors showed sensitivity to the chemotherapeutic agents paclitaxel [30] and carboplatin [31]. Therefore, we tested these agents on transplantable KPB1 tumors to demonstrate their utility as preclinical testing platforms (KPB1A and KPB1B; prior descriptions in Fig. 1b, tumors marked ‘chemo trial’ in Fig. 4a). For comparison, we tested a basal-like transplantable p53^−/−^ line (2225L) [14]. After tumor transplantation, mice bearing 5 mm tumors were randomized into control (no treatment) and treatment groups (carboplatin at 50 mpk in combination with paclitaxel at 10 mpk). As shown in Fig. 5a, chemotherapy regimens significantly extended overall survival in KPB1 transplant lines (*p* < 0.0001), while the p53^−/−^ 2225L line rapidly progressed to end stage (20 mm diameter). Observing the 14-day acute response, KPB1 tumors showed significant (*p* < 0.0001) regression in response to therapy while no regression from chemotherapy was observed in the 2225L p53^−/−^ line (Fig. 5b).

**Fig. 6:**
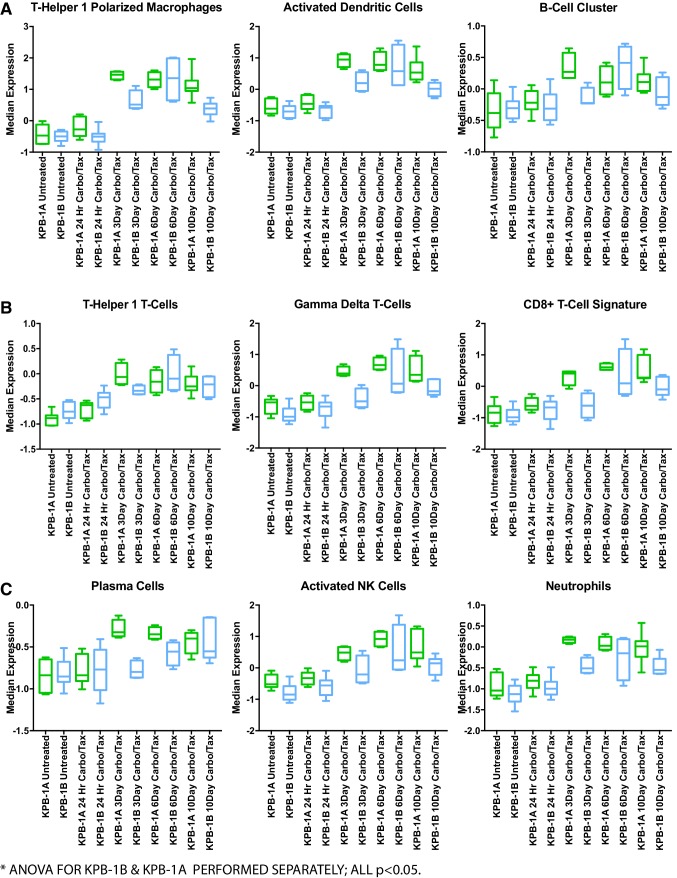
Carboplatin–paclitaxel combination therapy impacts immune cell signatures during response to therapy. **a** Immune cell signatures for cells typically associated with antigen presentation function are shown for KPB1A and KPB1B tumors across the specified therapeutic timepoints. Each immune cell signature is published as follows: Th1-polarized macrophages [32], activated dendritic cells [33], and the B-cell cluster signature [32]. **b** Immune cell signatures for T-cell subsets: Th1 T-Cells [26], Gamma-delta T-cells [33], and the CD8+T-cell signature [32]. **c** Other signatures for immune cells with anti-tumor functions are shown: plasma cells [33], activated NK cells [33], and neutrophils [33]. For each tumor line and time point, the sample sizes are as follows: KPB1A-no treatment *n* = 7, 24 h treated *n* = 10, 3 day treated *n* = 4, 6 day treated *n* = 4, and 10 day treated *n* = 7; KPB1B-no treatment *n* = 6, 24 h treated *n* = 10, 3 day treated *n* = 4, 6 day treated *n* = 4, and 10 day treated *n* = 7. Statistical analysis was conducted using an ordinary one-way ANOVA with KPB1A and KPB1B timepoints separately

Given the acute response to therapy, we examined the carboplatin/paclitaxel treated KPB1A and KPB1B tumors by gene expression analysis. Importantly, we selected timepoints during tumor regression to capture the transcriptomic changes associated with response to therapy. As expected, we observed a significant decrease in the proliferation signature post-therapy, with peak reduction observed at day 6 in both KPB1A and KPB1B (Fig. S3A, ANOVA *p* < 0.05). Changes in markers and signatures for immune cells were also prominent. We noted significant increases in signatures for cells capable of antigen presentation (Fig. 6a, ANOVA *p* < 0.05): **a** Th1-polarized macrophage signature, activated dendritic cell signature, and a signature for B-cells. In agreement, a significant increase in MHC class II genes occurred with therapy (Fig. S3B; ANOVA *p* < 0.05). In addition, Th1, gamma-delta, and CD8+T-cell signatures were significantly increased (Fig. 6b; ANOVA *p* < 0.05). Consistent with the cytotoxic effector functions of these types, we observed corresponding increases in interferon-gamma, Fas-ligand, TNF-alpha, and granzyme B (Fig. S3C–F, respectively; ANOVA *p* < 0.05). Signatures for other immune cell types were also increased (Fig. 6c). For example, we noted that a plasma cell signature was increased in both KPB1A and KPB1B lines (ANOVA *p* < 0.05). Interestingly, this signature exhibited an earlier increase in the expression in the KPB1A line (peaking at day 3) than in the KPB1B line (peaking at day 10). Examination of the IGG signature showed that KPB1B tumors had comparatively higher IGG expression at baseline, while KPB1A tumors increased IGG with course of therapy. Consistent with antibody-based immune cell activation, we also noted significant increases in signatures for NK cells and neutrophils (ANOVA *p* < 0.05). As a whole, these data suggest that chemotherapy increased both immune cell infiltration and the expression of genes associated with immune cell anti-tumor activity.

## Discussion

TP53 and BRCA1 loss in the mouse mammary gland results in tumors enriched for features of basal-like breast cancer. This model has a comparable genetic phenotype to a TP53/BRCA1 conditional mouse created by Liu et al. [34]. Similar to the Liu paper, we confirmed subtypes by histologic staining and verified them through a vigorous comparison using 27 distinct mouse models. We also found that these tumors have a large amount of genomic instability. The KPB1 mouse model not only clusters with other established basal-like mouse models but also serves as a model for identifying additional important pathways that are perturbed in basal-like breast cancers. The fact that over 400 copy number gains and losses each were conserved between the mouse and human basal-like cancers demonstrates that the KPB1 tumors are an ideal model for human basal-like breast cancer, and are even similar in terms of the propensity for each to have an immune cell infiltrate.

DawnRank and frequently observed copy number alterations common in murine and human basal tumors revealed genes that cooperate in the same signaling pathways. Importantly, each of these of the gains and losses corresponded to significant changes in gene expression signatures. For example, we observed frequent amplification of KRAS, AURKA, and MYC. Importantly, both AURKA and KRAS serve to stabilize and activate MYC signaling [35–37]. While a variety of studies have identified roles for other Wnt signaling factors in basal-like breast cancer [7, 38–40], few exist for WNT5B and TEAD4. Yet, recent work has shown that Wnt5B operates upstream of Tead4, stabilizing its coactivating protein Taz [41]. Cooperative events also occurred in Notch pathway genes NCSTN, PSEN1, and NOTCH4. The coordination of these genes in Notch signaling has been thoroughly reviewed [42] and Notch4 in particular has been shown to maintain breast cancer stem cells [43]. RB1 deletion and E2F1 amplification are frequent events in our KPB1 tumors. While E2F1 is well known to regulate cell cycle and tumor progression [44–46], E2F1 may also mediate basal-like features as loss of E2F1 reduced squamous tumor incidence [44]; importantly, squamous tumors show similarities to human basal-like breast cancer [18]. Together, the conservation between species, frequency, and cooperative nature of these events strongly suggest driver potential in basal-like tumors.

KPB1 tumors matched basal-like tumors for immune cell signatures and the nature of these signatures suggest KPB1 tumors to be immune-suppressed. This is evidenced by the elevation of T-regulatory cells, CTLA4 signaling, and Pd1 signatures. Th2 T-cell and B-cell signatures were also elevated in KPB1 and basal-like tumors. Suggesting interplay, Th2 T-cells are known to support B-cell proliferation [47]. Further, the elevated B-cell signatures were predominantly associated with immature B-cell states, which might relate to the elevation of T-regulatory cell signatures as T-regulatory cells have been shown to suppress B-cell activation [48, 49].

Despite the immune suppressive microenvironment of KPB1 tumors, paclitaxel/carboplatin combination therapy revealed the potential for adaptive immune responses. Unlike the p53^−/−^ basal-like tumor line (2225L), KPB1 tumors were sensitive to chemotherapy. This finding is expected as other Brca1 deficient models are sensitive to chemotherapy [50] and due to the type of DNA damage inflicted by carboplatin, which requires repair of this damage using homologous recombination-mediated DNA repair [51, 52]. However, the huge influx of immune cells during response to therapy was unexpected. With the specific immune signatures increased by chemotherapy, a possible mechanism of response may include cell death induced by chemotherapy and subsequent phagocytosis of cellular debris by antigen-presenting cells. This would allow for the presentation of tumor neoantigens to T-cells and thus further amplify the anti-tumor response. In support of this speculation, others have noted that immune-checkpoint therapy improves chemotherapy responses by activating adaptive immune cells [53]. In the future, the shared genomic and immune features between our KPB1 lines and human basal-like breast cancer will make this model valuable for investigating strategies to engage the immune system in treating basal-like breast cancer.

Because of the long tumor latency, we developed this GEMM into a syngeneic transplantation model. Both tumor transplant lines and tumor cell lines were created and credentialed with this study. Given the conservation of gene expression profiles and copy number alterations, we anticipate that these models will offer excellent translational value to investigations of human breast cancer. Therefore, this study provides the essential groundwork for tumor transplant lines and tumor cell lines that will be an important research tool for multiple studies focused on the nature of basal-like breast cancer, while at the same time identifying key driving pathways that spontaneously occur in both humans and mice.

## Electronic supplementary material

Below is the link to the electronic supplementary material.

Figure S1 Intrinsic analysis of mouse models and human breast cancers reveals basal-like gene expression profiles in K14-Cre; p53 ^f/f^ Brca1 ^f/f^ tumors. (A) Human breast cancer and GEMM co-cluster using an intrinsic gene list. Mouse and human tumors were preprocessed as described in the methods and ComBat was used to correct batch effects prior to gene filtering. Hierarchical clustering used centroid linkage; the dendrogram across the top depicts the relationship among tumor samples. Sky blue bars depict the position of luminal A tumors, navy blue bars depict the position of luminal B tumors, pink bars depict the position of Her-2 enriched tumors, red bars depict the position of basal-like tumors, green bars depict the position of normal-like tumors. Below the human annotations, murine tumors are annotated for their position in the dendrogram and in the heatmap below, with red bars depicting the position of KPB1 tumors and cell lines. All other mouse models are depicted with grey bars. Beside the heatmap, orange bars depict the position of clusters that we highlight in panel B. (B) Maintaining the cluster from panel A, we highlight individual clusters of genes that describe tumor subtype: i- basal-like genes, ii- luminal-like genes, iii-claudin low genes, iv- proliferation genes. All genes are expressed according to the green-black-red color bar. Supplementary material 1 (PDF 5250 KB)

Figure S2- Examination of immune-suppressive gene expression features amongst basal-like tumors. Box and whiskers plots of immune cell signatures across murine (KPB1-basal-like n=31, p53 basal-like n=33, Neu ^Ex^ n=36, and PyMT ^EX^ n=17) and human tumors (basal-like n=136 ; and luminal-like n= 591). (A) PDCD1 gene expression is shown, (B) PD-L1 gene expression is shown, and (C) a gene expression signature for CTLA4 signaling is shown (from molecular signatures database). T-tests were unpaired and two-tailed p-values are reported as follows : * p<0.05 for KPB1 model compared to the corresponding mouse model; ** p<0.05 for human basal-like versus luminal-like tumors. Supplementary material 2 (PDF 263 KB)

Figure S3 Carboplatin-paclitaxel combination therapy impacts proliferation and immune cell genes during response to therapy. (A) Expression patterns for the proliferation signature across treatment time points. (B) Expression patterns for the MHC Class II genes across treatment timepoints. (C) Expression of the interferon gamma gene across treatment timepoints. (D) Expression of the TNF-alpha gene across treatment timepoints. (E) Expression of the Fas-ligand gene across treatment timepoints. (F) Expression of the granzyme B gene across treatment timepoints. (G) Expression of the IGG gene signature across treatment timepoints. For each tumor line and time point the sample sizes are as follows: KPB1A- no treatment n=7, 24 hour treated n= 10, 3 day treated n= 4, 6 day treated n= 4, and 10 day treated n=7; KPB1B- no treatment n=6, 24 hour treated n= 10, 3 day treated n= 4, 6 day treated n= 4, and 10 day treated n=7. Statistical analysis was conducted using an ordinary one-way ANOVA with KPB1A and KPB1B timepoints separately. Supplementary material 3 (PDF 361 KB)
