# Supplementary figures and images for: A mouse model featuring tissue-specific deletion of p53 and Brca1 gives rise to mammary tumors with genomic and transcriptomic similarities to human basal-like breast cancer
Source: Breast Cancer Res Treat. 2018 Nov 27;174(1):143–55. doi: 10.1007/s10549-018-5061-y (PMC6418066; doi:10.1007/s10549-018-5061-y)

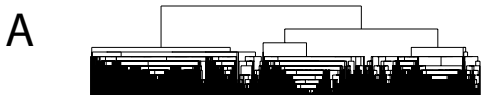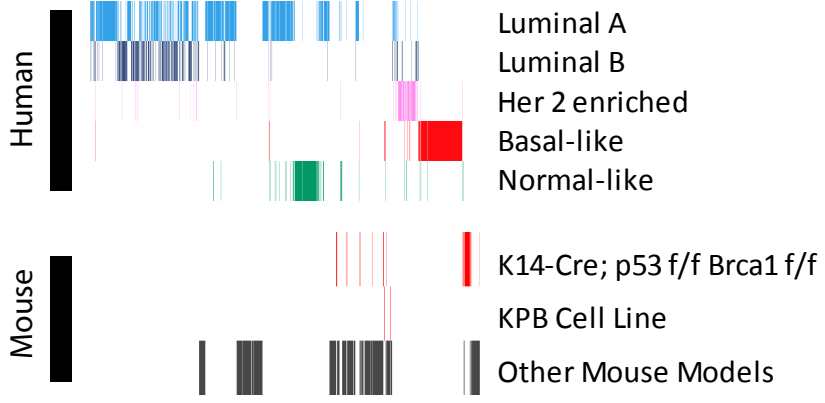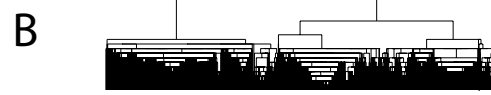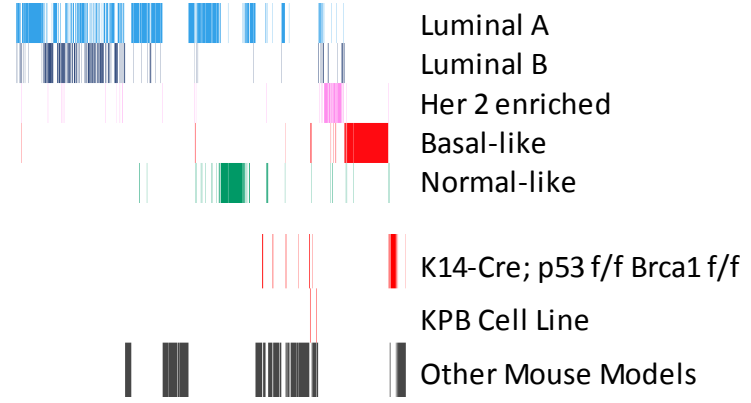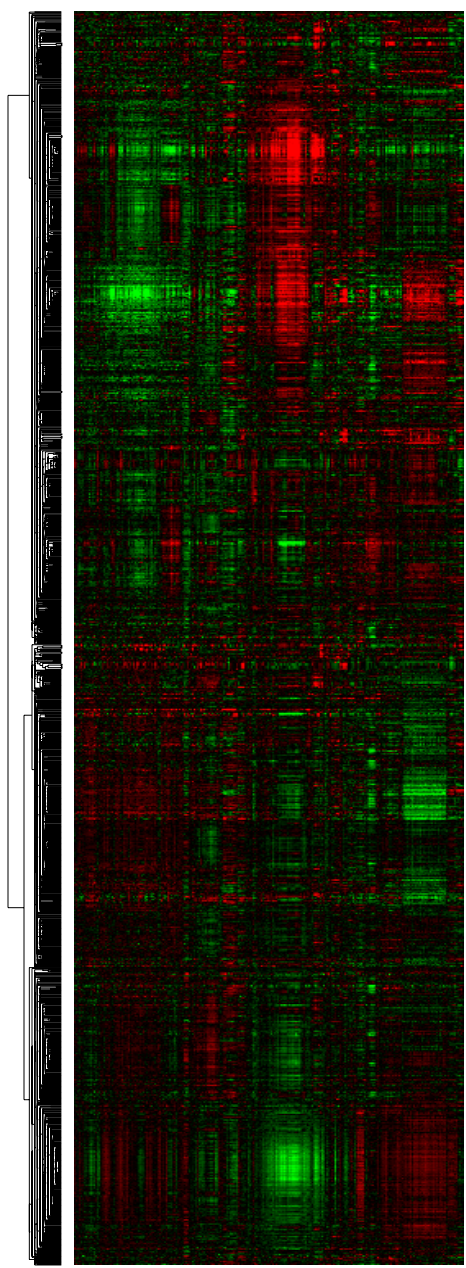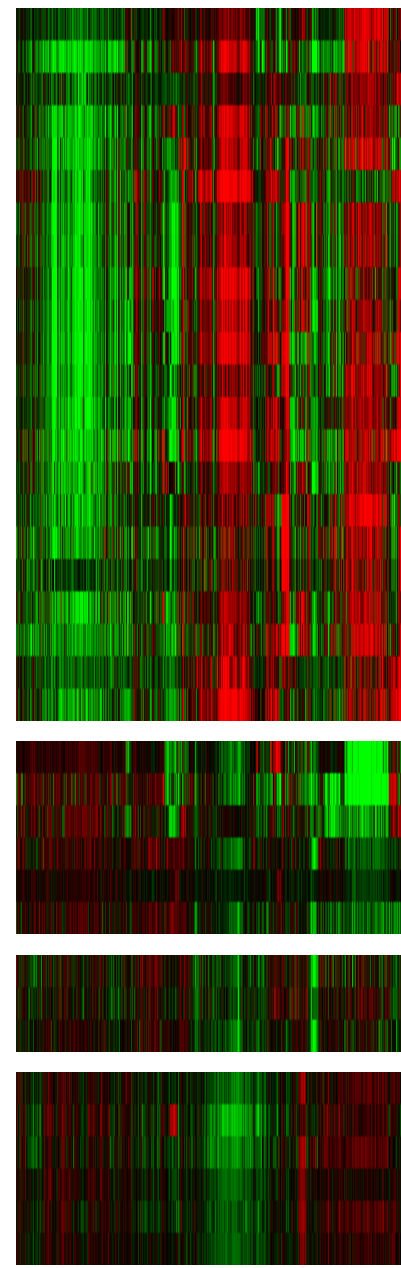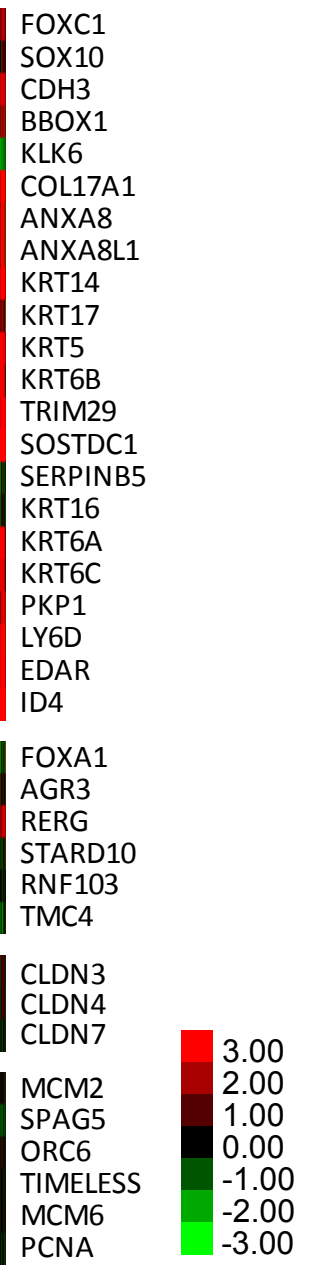

Supplement: Supplementary file 1 — Figure S1 Intrinsic analysis of mouse models and human breast cancers reveals basal-like gene expression profiles in K14-Cre; p53 f/f Brca1 f/f tumors. (A) Human breast cancer and GEMM co-cluster using an intrinsic gene list. Mouse and human tumors were preprocessed as described in the methods and ComBat was used to correct batch effects prior to gene filtering. Hierarchical clustering used centroid linkage; the dendrogram across the top depicts the relationship among tumor samples. Sky blue bars depict the position of luminal A tumors, navy blue bars depict the position of luminal B tumors, pink bars depict the position of Her-2 enriched tumors, red bars depict the position of basal-like tumors, green bars depict the position of normal-like tumors. Below the human annotations, murine tumors are annotated for their position in the dendrogram and in the heatmap below, with red bars depicting the position of KPB1 tumors and cell lines. All other mouse models are depicted with grey bars. Beside the heatmap, orange bars depict the position of clusters that we highlight in panel B. (B) Maintaining the cluster from panel A, we highlight individual clusters of genes that describe tumor subtype: i- basal-like genes, ii- luminal-like genes, iii-claudin low genes, iv- proliferation genes. All genes are expressed according to the green-black-red color bar. Supplementary material 1 (PDF 5250 KB) [file 10549_2018_5061_MOESM1_ESM.pdf]

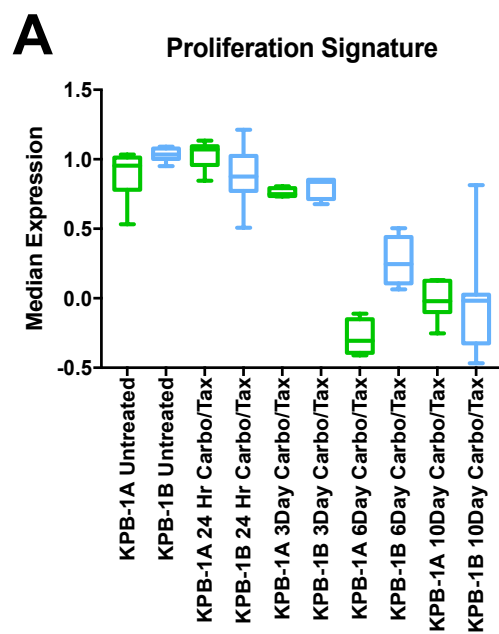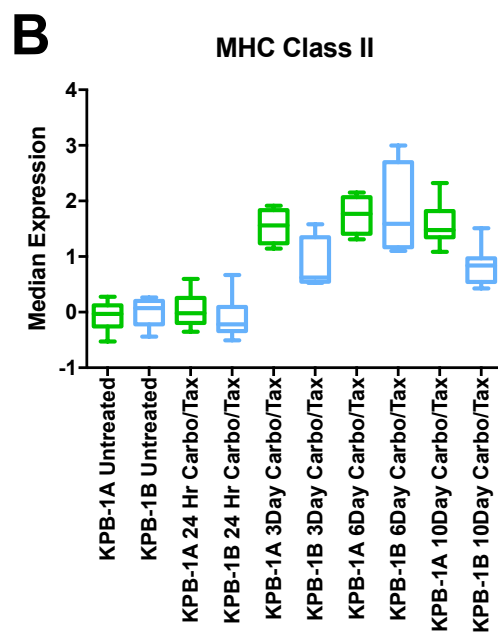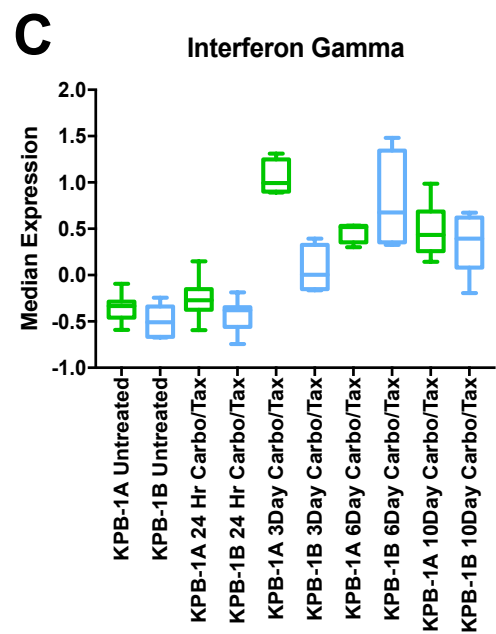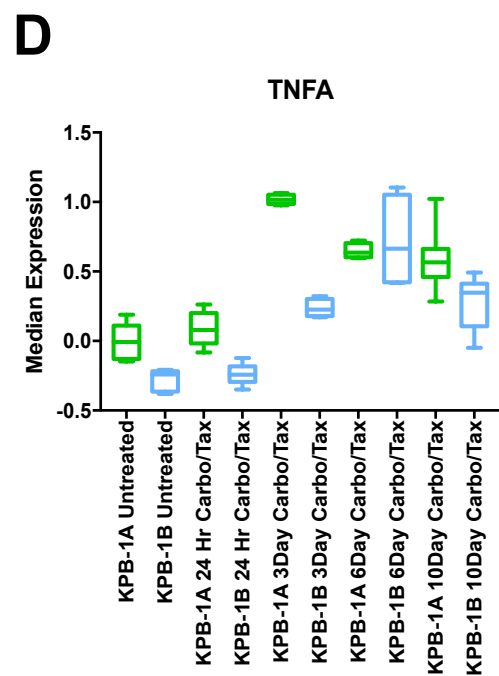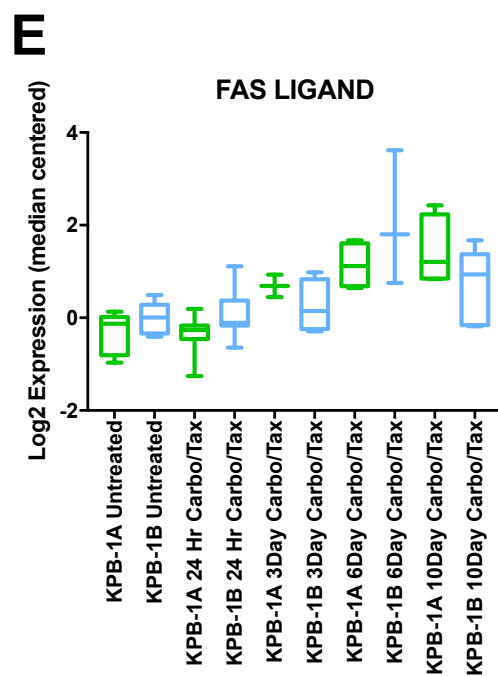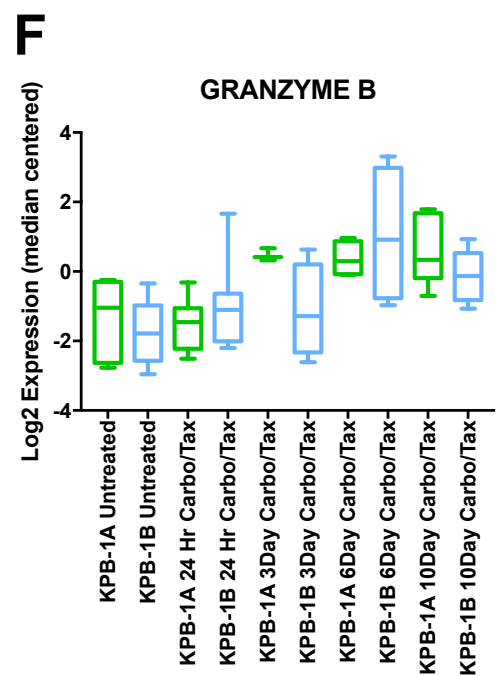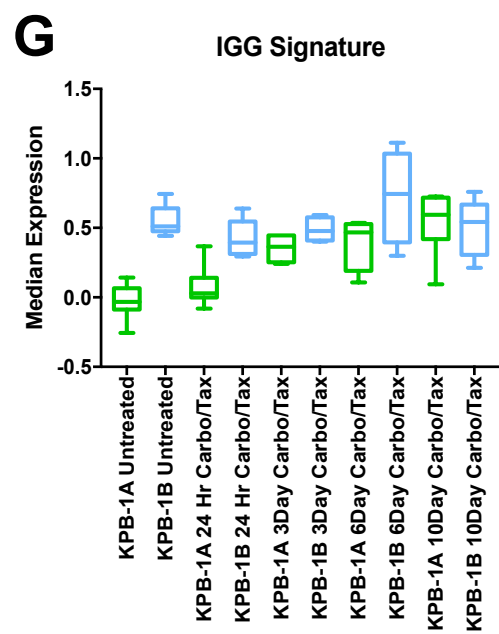

\* ANOVA FOR KPB-1B & KPB-1A PERFORMED SEPARATELY; ALL  $p < 0.05$ .  
EXCEPTION: IGG Signature  $p = 0.1$  in KPB-1B line.

Supplement: Supplementary file 3 — Figure S3 Carboplatin-paclitaxel combination therapy impacts proliferation and immune cell genes during response to therapy. (A) Expression patterns for the proliferation signature across treatment time points. (B) Expression patterns for the MHC Class II genes across treatment timepoints. (C) Expression of the interferon gamma gene across treatment timepoints. (D) Expression of the TNF-alpha gene across treatment timepoints. (E) Expression of the Fas-ligand gene across treatment timepoints. (F) Expression of the granzyme B gene across treatment timepoints. (G) Expression of the IGG gene signature across treatment timepoints. For each tumor line and time point the sample sizes are as follows: KPB1A- no treatment n=7, 24 hour treated n= 10, 3 day treated n= 4, 6 day treated n= 4, and 10 day treated n=7; KPB1B- no treatment n=6, 24 hour treated n= 10, 3 day treated n= 4, 6 day treated n= 4, and 10 day treated n=7. Statistical analysis was conducted using an ordinary one-way ANOVA with KPB1A and KPB1B timepoints separately. Supplementary material 3 (PDF 361 KB) [file 10549_2018_5061_MOESM3_ESM.pdf]
