# Supplementary material for: A mouse model featuring tissue-specific deletion of p53 and Brca1 gives rise to mammary tumors with genomic and transcriptomic similarities to human basal-like breast cancer
Source: Breast Cancer Res Treat. 2018 Nov 27;174(1):143–55. doi: 10.1007/s10549-018-5061-y (PMC6418066; doi:10.1007/s10549-018-5061-y)
Supplement: Supplementary file 2 — Figure S2- Examination of immune-suppressive gene expression features amongst basal-like tumors. Box and whiskers plots of immune cell signatures across murine (KPB1-basal-like n=31, p53 basal-like n=33, Neu Ex n=36, and PyMT EX n=17) and human tumors (basal-like n=136 ; and luminal-like n= 591). (A) PDCD1 gene expression is shown, (B) PD-L1 gene expression is shown, and (C) a gene expression signature for CTLA4 signaling is shown (from molecular signatures database). T-tests were unpaired and two-tailed p-values are reported as follows : * p<0.05 for KPB1 model compared to the corresponding mouse model; ** p<0.05 for human basal-like versus luminal-like tumors. Supplementary material 2 (PDF 263 KB) [file 10549_2018_5061_MOESM2_ESM.pdf]

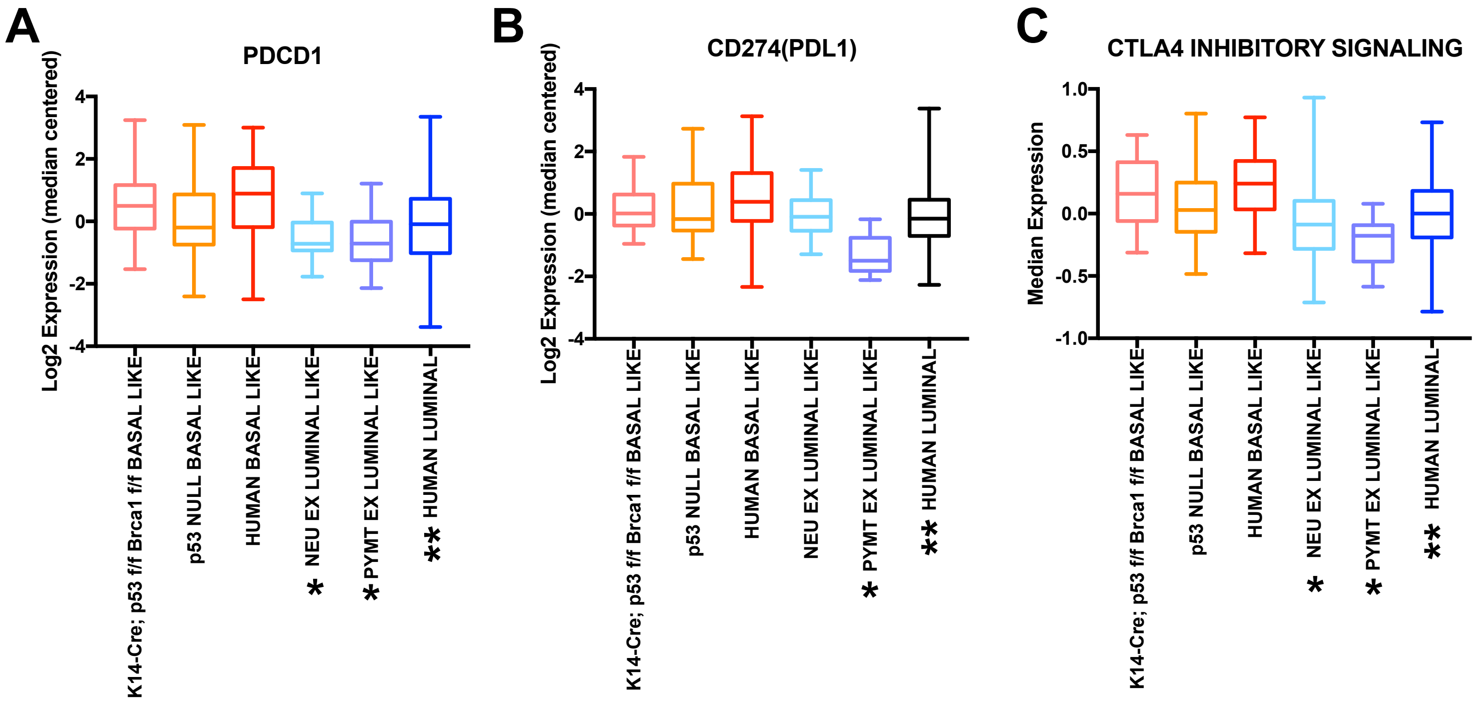

* = $p<0.05$ in murine comparison to K14-Cre; p53 f/f Brca1 f/f .

** = $p<0.05$ in comparison of human basal-like vs luminal tumors.
